# Pulmonary and Physical Virtual Reality Exercises for Patients With Blunt Chest Trauma: Randomized Clinical Trial

**DOI:** 10.2196/54389

**Published:** 2024-12-09

**Authors:** Tjitske D Groenveld, Indy GM Smits, Naomi Scholten, Marjan de Vries, Harry van Goor, Vincent MA Stirler

**Affiliations:** 1 Department of Surgery Radboud University Medical Center Nijmegen Netherlands; 2 Department of Rehabilitation Radboud University Medical Center Nijmegen Netherlands; 3 Kromhout Kazerne Ministry of Defence Military Health Organisation Utrecht Netherlands

**Keywords:** virtual reality, pain, pulmonary, chest trauma, blunt thorax trauma, pain relief, breathing, mobilization, randomized clinical trial, clinicians, rehabilitation, physical activity, exercise, interview

## Abstract

**Background:**

Adequate pain relief, early restoration of breathing, and rapid mobilization pose a clinical challenge in patients with blunt chest trauma. Virtual reality (VR) has the potential to achieve these 3 interrelated treatment objectives with enhanced self-efficacy and autonomy of patients and limited support by clinicians.

**Objective:**

This study aimed to assess the effectivity of breathing and physical exercises using VR on the pulmonary recovery of patients with blunt chest trauma at the ward.

**Methods:**

A pilot randomized controlled trial was performed. The control group received usual physiotherapy consisting of protocolized breathing exercises (8 times daily for 10 minutes) and physical exercises (2 times daily for 10 minutes). The VR group was instructed to perform these exercises using VR. The primary outcome was vital lung capacity at day 5 or earlier at discharge. Secondary outcomes were patient mobility (time standing, lying, and sitting), clinical outcomes (length of hospital stay, pulmonary complications, transfer to intensive care unit, and readmission within 30 days), pain, activities of daily living, patient-reported outcome measures (satisfaction and quality of recovery). Patient experiences and barriers and facilitators toward implementation were assessed through interviews.

**Results:**

The study was prematurely ended due to enrollment failure combined with poor protocol adherence to exercises in both groups. A total of 27 patients were included, of which 19 patients completed 3 or more days. Vital lung capacity at 5 days (or last measurement) was equal between groups with 1830 (SD 591) mL and 1857 (SD 435) mL in the control and VR groups, respectively. No marked differences were observed in secondary outcomes. Patient interviews showed positive attitudes toward the use of VR, describing that visualization of the exercises helped patients to perform the exercises correctly and to continue the exercises for a longer duration. Also, patients experienced the immersiveness of VR as an analgesic. However, patients did not experience added value over usual care and reported that better integration in treatment and the hectic hospital environment could improve the use of the VR exercises.

**Conclusions:**

The suitability of patients to use virtual reality therapy (VRx) in a hospital (trauma) ward setting is lower than generally expected. Effective application of VRx requires professional guidance and needs thorough alignment with clinical practice. For future research, we recommend to chart adherence to study protocol before designing a VR clinical trial. Patient-reported experiences need to be prioritized in evaluating VR acceptance, usability, and effectiveness. In line, we recommend performing a systematic analysis (eg, using the technology acceptance model) on the acceptance before pilot or main effectiveness studies. Finally, the eligibility of patients and exclusion of patients due to the inability to use VRx should be routinely reported.

**Trial Registration:**

ClinicalTrials.gov NCT05194176; https://tinyurl.com/2bzh4tzx

## Introduction

Blunt chest trauma comprises over 10% of all trauma patients presenting to emergency departments worldwide and is the most frequent injury (44.5%) in patients with multiple traumas [1-3]. The most frequent injuries are rib fractures, pneumothorax, and pulmonary contusion [2,4]. Chest trauma is associated with high risk (>10%) of pulmonary complications such as pneumonia, acute respiratory distress syndrome, and need for ventilatory support [5-7]. Mortality after blunt chest trauma is 4%-20%, with pneumonia being the most important risk factor [1,6,8].

Management is mainly focused on the prevention of these pulmonary complications. The most important pillars herein are adequate pain relief, breathing exercises, and rapid mobilization [6]. These treatment objectives are interrelated and commonly addressed in care bundles. Care bundles evidently improve clinical outcomes and decrease intensive care unit (ICU) and hospital length of stay [9,10]. Inadequate pain control can result in restricted ventilatory function and reduced mobility, both adding to a higher risk of complications. Currently, multimodal analgesics (different combinations of epidural analgesics, opioids, nonsteroidal anti-inflammatory drugs, and so on) are recommended for pain relief [11]. Epidural and systemic opioids are the most frequently used modalities [10]. However, especially opioids can have deleterious side effects such as sedation and hypoventilation, which directly negatively affect pulmonary function recovery. In addition, side effects, such as nausea and dizziness, can refrain patients from physical activity [12]. As a result, effective pain control without disrupting pulmonary recovery remains a challenge in daily clinical practice. Furthermore, physiotherapists play an important role in the prevention of complications, supporting patients with breathing and physical exercises. Breathing exercises are delivered by physiotherapists and address the active cycle of breathing technique and deep breathing [9,13]. Physical exercises mainly focus on maintaining and restoring the active range of motion of the trunk and limbs and restoring independence in functional activities [13]. Higher levels of physical exercise are associated with better functional outcomes and reduced length of hospital stay, whereas inactivity can result in general deconditioning and subsequent complications [14]. Previous reports show that hospitalized trauma patients spend between 53%-57% of the time lying in bed, despite interventions to improve physical activity [15,16]. Besides promoting general activity, physical exercises specifically aim at maintaining and restoring the active range of motion of the trunk and limbs and independence in functional activities [13].

Therapeutic virtual reality (VR) has the potential to address the 3 treatment objectives (pain relief, adequate breathing, and rapid mobilization) in blunt chest trauma [17-19]. The ability of VR to immerse a person in another world presents various opportunities, including the reduction of acute pain and procedural pain, helping patients to relax and breathe normally, and motivating them to engage in more physical exercise [17,20,21]. VR has proven effective for reducing procedural pain by distracting patients from the painful experience through an immersive and playful environment [20]. Exergaming, gaming that requires physical exercise, has shown to improve patient adherence and physical fitness [18]. Furthermore, virtual reality therapy (VRx) in a hospital setting has shown to reduce pain, anxiety, and possibly length of stay [22]. Another advantage of VRx is the few side effects, which are usually mild and transient [23].

In this study, the primary aim was to assess the effectivity of breathing and physical exercises using VR on the pulmonary recovery of patients with blunt chest trauma at the ward. Our hypothesis was that patients would experience faster pulmonary recovery due to a better execution of the exercises with use of VR. Secondary aims were to assess patient mobility, clinical outcomes, pain, and patient-reported outcomes and experiences. Assessment of patient experiences, attitudes, and expectations is relevant because VR treatment for this indication is new and not routine at hospital wards.

## Methods

### Design

This was an open-label randomized controlled trial conducted between March 2022 and January 2023 at Radboud University Medical Center, Nijmegen, the Netherlands. The trial is reported according to the CONSORT (Consolidated Standards of Reporting Trials) guidelines for randomized trials (Multimedia Appendix 1) [24].

### Ethical Considerations

Ethical approval was obtained by the Research Ethics Committee of the Radboud University Medical Centre, the Netherlands (Commissie Mensgebonden Onderzoek Arnhem-Nijmegen, NL80011.091.21). Eligible patients were approached by their treating physician or nurse and received verbal and written information about the study. A member of the research team answered any additional questions and obtained informed consent. All participants received a unique research ID linked to their personal details. The participants’ data were securely stored on the institution’s server, and access to the specific folders was restricted to the researchers involved in the study. The study data were stored using only the research ID, with no personal identifiers, in Castor Electronic Data Capture (Amsterdam, the Netherlands). Participants received no reimbursement. This trial was conducted according to the principles of the Helsinki Declaration and in accordance with Dutch guidelines, regulations, and Acts (Medical Research involving Human Subjects Act, WMO).

### Participants

The study population comprised of patients aged 16 years and older, with no upper age limit, who had a blunt chest trauma and were directly admitted to the trauma and orthopedic ward from the emergency department. The study included all genders to account for potential variations in pulmonary mechanics. Exclusion criteria were (1) neurotrauma with Glasgow Coma Scale of 13 and lower; (2) history of dementia, seizures, and epilepsy; (3) significant hearing or visual impairment which is not corrected; (4) headwounds or damaged skin with which comfortable and hygienic wear of head-mounted display (HMD) is not possible; (5) stay at ICU during current hospital admission for reasons other than observation or a duration of 48 hours or longer; and (6) erect position in bed is not possible or allowed. Patients were recruited on the first day of their admission. Patients were subsequently randomized (1:1) to the VR group or control group using computer-generated block randomization.

### Intervention

All patients from both groups received care according to the existing guideline [25]. Similarly, pain management was according to the World Health Organization guidelines for acute pain in adults [26]. Once daily, the control group received usual physiotherapy consisting of breathing and physical exercises. Breathing exercises include deep breathing, huffing, and coughing following the active cycle of breathing technique. Patients were instructed by the physiotherapist and received a leaflet with written instructions. Physical exercises include practice of functional movement (eg, activities of daily living) and exercises for range of motion of the trunk and limbs. Patients were instructed to perform breathing exercises 8 times daily for 10 minutes and to extend these exercises 2 times daily with (sitting) physical exercises for an additional 10 minutes.

The VR group was instructed to perform the breathing exercises using the VR intervention 8 times daily for 10 minutes and to extend these exercises 2 times daily with (sitting) physical exercises using VR for an additional 10 minutes. For all VR exercises an HMD, the PICO Neo 3 (Pico) was used. The commercially available apps SyncVR Fit and SyncVR Relax & Distract (SyncVR Medical) were used. This selection of apps was made in consultation with physiotherapists and nurses to ensure that the games were suitable and beneficial for the patients. This collaborative approach aimed to choose games that would effectively address the therapeutic objectives of pain relief, adequate breathing, and rapid mobilization. SyncVR Fit contains breathing exercises that are comparable to the exercises in usual physiotherapy care but can be performed in a virtual environment. The physical VR exercises consist of several games through which patients are challenged to reach out to objects while involving their arms, head, and trunk. Once daily, the exercises were performed under the supervision of a physiotherapist, the other sessions were unsupervised. SyncVR Relax & Distract contains several mindfulness and relaxing exercises to support in coping with pain and anxiety. Patients were allowed to use these at their own discretion in addition to the prescribed exercises with a maximum of 30 minutes per VR session to prevent side effects.

In both groups, a research team member visited the patients twice daily to perform measurements. At these moments, patients were encouraged to perform the exercises in attendance of the research team member. In the VR group, this was primarily to detect any technological difficulties and lower the bar for patients to use the VR headset. The control group was visited accordingly to prevent bias.

### Outcome Measures

Primary outcome was the vital lung capacity (in mL), measured using an incentive spirometer (IS; Voldyne [Teleflex Medical]), on day 5 of the study or the last measurement between 3 and 5 days. Incentive spirometry was chosen over usual spirometry despite lower reliability because transferring patients to a spirometer at least once daily would have required significant logistical support and funding. In addition, it would have imposed an additional burden on both patients, who were sometimes quite ill, and the already busy personnel. To ensure consistency in the measurements, a standardized protocol was followed, which included recording the best score out of 3 attempts. Secondary outcomes were patient mobility, analgesics use, clinical outcomes, pain, activities of daily living, patient-reported outcome measures, safety outcomes, and barriers and facilitators toward implementation. Patient mobility was defined as the percentage of time spent lying, sitting, and moving measured using a wearable activity monitor (activPAL3 [Pal Technologies Ltd]). The type and dosage of analgesics used were extracted from patient files for the first 5 days of the study. Opioid use was calculated and reported as oral morphine equivalent. Clinical outcomes included the length of hospital stay (in days), pulmonary complications during admission, transfer to ICU, and readmission within 30 days. Pain was assessed during breathing exercises using a visual analog scale (VAS) with 0 being no pain and 10 being extreme pain. Activities of daily living were measured using the powerlessness in daily living (PDL) questionnaire, with lower scores representing more independence in activities of daily living [27]. Patient-reported outcomes and experiences comprised the Quality of Recovery-15 (QoR-15) questionnaire [28], modified treatment satisfaction questionnaire (MTSQ) [29], and semistructured interviews regarding patients’ satisfaction and experiences with VR. Reasons for withdrawal and side effects during VRx were registered as safety outcomes. Barriers and facilitators were derived from the semistructured interviews and patient diaries in which patients reported on treatment adherence, technical problems, and feedback on the VR exercises.

### Study Procedures

The duration of the study for patients was 5 days from the moment of inclusion on the first day of their admission or less when the patient was discharged earlier. After randomization, participants in the intervention group received instructions from a research team member on how to use the VR intervention, and a supervised training session was performed. If needed, additional technical support was provided by the research team. Patient characteristics were extracted from electronic patient files and additional characteristics were asked upon inclusion. A wearable activity monitor was installed on the first day. At baseline IS, VAS pain score, and PDL score were measured. During days 2-5, daily IS, VAS pain score during breathing exercises, PDL score, and patient mobility were registered for both groups. IS was measured twice daily following a standardized procedure. Patients were instructed to register the frequency of pulmonary and physical exercises in a daily diary as well as to fill out the quality of recovery questionnaire and any experienced side effects (open-ended question). On day 5, participants received the treatment satisfaction questionnaire. Patients in the intervention groups that used the VR intervention at least once were asked on day 5 to participate in a short semistructured interview with the predetermined topics, such as experiences with VR regarding efficacy and barriers and facilitators using VR from patients’ perspectives. After 5 days, participants in the VR group were allowed to choose to continue the VR intervention or to return to usual care. Patients were followed up for 30 days after discharge to register clinical outcomes such as late complications. In Multimedia Appendix 2, all study procedures are schematically presented.

### Sample Size

The calculated needed sample size was 63 patients per group. This calculation was based on the primary outcomes measure vital lung capacity. The mean outcome of the control group was set on 1250 cc and SD was set on 500 cc based on the literature [30]. The mean outcome of the VR group was based on an expected 20% (clinically relevant) improvement, resulting in 1500 cc. An α of .05 and β of .20 were applied.

### Statistical Analyses

Quantitative analyses were done using IBM SPSS Statistics (version 25). Qualitative analyses were done using Atlas.ti (version 23.0.7; Lumivero). Descriptive statistics were used to present quantitative data. For effectivity outcomes, only patients who completed at least 3 study days were eligible for evaluation. No significance was calculated due to the low number of participants. For safety outcomes (eg, side effects), all patients were evaluated. Mobility data derived from the activPAL devices are automatically checked for validity by built-in algorithms following the validation criteria of PAL technologies [31]. This validity is indicated per measurement day, meaning that all measurements for a given day are regarded as either valid or invalid. This applies uniformly to all mobility measures, including standing, lying or sitting, and walking. Only valid wear days were evaluated.

The content of the interviews was transcribed ad verbatim and analyzed in Atlas.ti (version 23) using inductive thematic analysis. To minimize bias, 2 independent researchers with a background in medicine performed thematic analysis. Transcripts were coded according to the predefined main topics. Identified codes were categorized into themes and discussed until a consensus was reached.

## Results

### Overview

**Figure 1:**
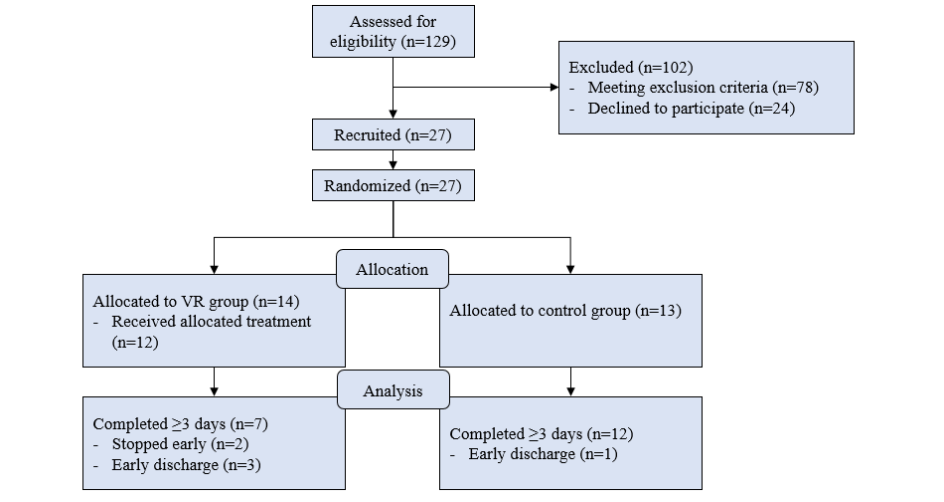
Study flowchart.

Between March 2022 and February 2023, a total of 129 patients were screened for eligibility and 27 patients were recruited (Figure 1). Reasons for exclusion are described in Table 1. In the VR group, 2 patients did not receive the intervention; 1 patient withdrew before the intervention was started, and 1 patient was excluded on day 2 due to previously unrecognized cognitive impairments. In the VR group, 5 patients did not complete 3 days; 1 patient stopped early because he found the exercises too time demanding, 1 patient stopped because she was unable to follow study protocol, and 3 patients in the VR group were nonevaluable due to discharge before completing 3 study days. In total, 19 patients were evaluable (completed 3 or more days) of which 7 in the intervention and 12 in the control group. In total, 4 patients in the intervention group and 8 patients in the control group completed the full 5 days of the study protocol. Majority of evaluable patients were male (15/19, 79%). The participants’ mean age was 60 years. Mean Injury Severity Score was 15 (SD 7; Table 2). Adherence to exercises was poor in both groups, with an average of performing breathing exercises 2 times per day in the VR group and 3 times per day in the control group (Table 3).

**Table 1:** Reasons for exclusion of patients assessed for eligibility.

Reason		Patients excluded, n
**Based on exclusion criteria**		78
	Stay at intensive care unit during current hospital admission, for reasons other than observation or a duration of >48 hours	33
	Expected discharge within 24 hours (not able to comply with study protocol)	18
	Headwounds or damaged skin with which comfortable and hygienic use is not possible.	11
	Not able to comply with study protocol (eg, language)	9
	History of dementia, delirium, seizures, and epilepsy	4
	Glasgow Coma Scale ≤13	3
**Declined to participate**		16
	Patient feels too sick	4
	Patient finds participation too burdensome	5
	Patient has too much pain	1
	Patient feels like having too less experience with technical devices	1
	Disinterest	5
Other or unknown		8

**Table 2:** Patient and disease characteristics.

Characteristic		VR group			Control group	
		All (n=14)	Evaluable (n=7)	All (n=13)		Evaluable (n=12)
				
Age (years), mean (SD)		63 (10)	62 (10)	55 (16)		56 (17)
Male participants, n (%)		11 (79)	6 (86)	10 (77)		9 (75)
**Education, n (%)**						
	ISCED^a^ 2^b^	6 (43)	3 (43)	4 (31)		4 (33)
	ISCED 3-4^c^	4 (29)	2 (29)	5 (39)		5 (42)
	ISCED 5-6^d^	1 (7)	1 (14)	0 (0)		0 (0)
	ISCED 7^e^	2 (14)	1 (14)	4 (31)		3 (25)
	Missing	1 (7)	0 (0)	0 (0)		0 (0)
**Occupation, n (%)**						
	Health care	0 (0)	0 (0)	2 (15)		2 (17)
	Trade and services	5 (36)	3 (43)	6 (46)		5 (42)
	Agriculture and nature	1 (7)	0 (0)	0 (0)		0 (0)
	Media and communication	0 (0)	0 (0)	1 (8)		1 (8)
	Education and culture	2 (14)	1 (14)	0 (0)		0 (0)
	Engineering and construction	4 (29)	2 (29)	2 (15)		2 (17)
	Other	1 (7)	1(14)	2 (15)		2 (17)
	Missing	1 (7)	0 (0)	0 (0)		0 (0)
**Smoking, n (%)**						
	No	5 (36)	3 (43)	10 (77)		9 (75)
	Yes	4 (29)	3 (43)	1 (8)		1 (8)
	Stopped	4 (29)	1 (14)	2 (15)		2 (17)
History of pulmonary disease (yes), n (%)		2 (14)	0 (0)	1 (8)		1 (8)
Chronic pain (yes), n (%)		5 (36)	1 (14)	4 (31)		4 (33)
**Trauma mechanism, n (%)**						
	Motor vehicle accident	2 (14)	1 (14)	0 (0)		0 (0)
	Fall from height	3 (21)	3 (42)	2 (15)		2 (17)
	Motorcycle >20 km/h	2 (14)	0 (0)	0 (0)		0 (0)
	Bike accident >20 km/h	3 (21)	1 (14)	6 (46)		6 (50)
	Bike accident <20 km/h	2 (14)	1 (14)	2 (23)		2 (17)
	Domestic fall	2 (14)	1 (14)	1 (8)		1 (8)
	Other	0 (0)	0 (0)	1 (8)		1 (8)
**Type of chest trauma, n (%)**						
	Rib fracture	14 (100)	7 (100)	13 (100)		12 (100)
	Pneumothorax	8 (57)	4 (57)	4 (31)		3 (25)
	Hemothorax	2 (14)	2 (29)	2 (15)		2 (17)
	Sternum fracture	1 (7)	0 (0)	2 (15)		2 (17)
	Lung contusion	6 (43)	3 (43)	1 (8)		1 (8)
**Concurrent trauma, n (%)**		—^f^	7 (100)	—		8 (67)
	Neurotrauma	4 (29)	4 (57)	1 (8)		1 (8)
	Upper extremity	7 (50)	4 (57)	5 (39)		5 (42)
	Lower extremity	2 (14)	0 (0)	2 (15)		2 (17)
	Internal organ damage	5 (36)	2 (29)	3 (23)		3 (25)
	Pelvic damage	3 (21)	0 (0)	1 (8)		1 (8)
	Spine	3 (21)	2 (29)	4 (31)		4 (33)
Injury Severity Score, mean (SD)		19 (7)	17 (8)	13 (6)		14 (6)
Operative management of chest trauma, n (%)		1 (7)	1 (14)	1 (8)		1 (8)
**Use ≥3 digital technologies, n (%)**						
	Daily	7 (50)	4 (57)	10 (77)		10 (83)
	Weekly	10 (71)	6 (86)	13 (100)		12 (100)
Experience with virtual reality (yes)		1 (8)	1 (14)	5 (39)		4 (33)

^a^ISCED: International Standard Classification of Education.

^b^ISCED 2: lower secondary.

^c^ISCED 3-4: upper-secondary to postsecondary nontertiary education.

^d^ISCED 5-6: short-cycle tertiary education, Bachelor’s, or equivalent level.

^e^ISCED 7: master’s degree.

^f^Not applicable.

**Table 3:** Average number of breathing and physical exercises performed by patients who completed 3 or more study days.

Intervention group		Day 2, mean (SD)	Day 3, mean (SD)	Day 4, mean (SD)	Day 5, mean (SD)
**VR^a^ group^b^**					
	Breathing	2.6 (1.6)	1.6 (2.1)	2 (2.9)	2 (2.8)
	Physical	1.3 (0.7)	1.1 (1.0)	0.6 (1.2)	0.8 (0.8)
**Control group^c^**					
	Breathing	4.0 (2.5)	3.8 (3.1)	3.5 (3.2)	2.1 (2.6)
	Physical	1.4 (1.7)	1.9 (2.3)	1.3 (2.5)	1.8 (2.1)

^a^VR: virtual reality.

^b^For days 2-4, n=7; for day 5, n=5.

^c^For days 2 and 3, n=12; day 3, n=10; and day 5, n=8.

### Primary Outcome

Vital lung capacity at 5 days (or last measurement between day 3 and 5) was similar between groups with 1830 (SD 591) mL in the control group and 1857 (SD 435) mL in the VR group. The vital lung capacity over the days improved with an average of 421 (SD 345) mL between day 1 and 3 in the VR group followed by a decrease of 208 (SD 315) mL between day 3 and 5, which is likely due to early discharge of rapidly recovered patients (Multimedia Appendix 3). In the control group, an average improvement of 389 (SD 370) mL was observed between day 1 and 3 with a further increase of 246 (SD 292) mL between days 3 and 5.

### Secondary Outcomes

Regarding the mobility data measured using activPal, 58% (24/37; SD 36) of measurement days was valid in the control group and 61% (13/21; SD 34) was valid in the VR group. Mobility was similar between groups (Multimedia Appendix 3) with majority of time spent sitting (in bed) or lying. Patients were standing or walking for only 3%-5% of the time in both groups on all days. Analgesics use was similar between groups (Multimedia Appendix 4). In both groups, there was a slight improvement in the VAS pain score during breathing exercises over the course of 3 to 5 days (Multimedia Appendix 3). For the PDL questionnaire, a similar pattern was found as for vital lung capacity with initial improved median scores in the VR group from 9 (IQR 8-12) points to 5.0 (IQR 4-5) points between day 1 and 3 and from 11 (IQR 4-15) to 4 (IQR 0-13) in the control group within both groups worsening between day 3 and 5 (Multimedia Appendix 3). Regarding the quality of recovery, the control group scored median daily scores between 83.0 (IQR 76-97) and 99 (IQR 89-111) compared with 81 (IQR 57-111) and 108 (IQR 67-110) in the VR group (Multimedia Appendix 3). Mean global satisfaction was 72.9 (SD 12.5) points on a scale from 0 to 100 in the VR group versus 65.6 (SD 15.7) in the control group. Median length of hospital stay was 5 days in the VR group and 6 days in the control group. No pulmonary complications, ICU admissions, or readmission within 30 days were observed in both groups.

### Interviews

A total of 6 patients of the VR group were interviewed—4 males and 2 females. Mean age was 57 years. Interviews were conducted face-to-face or by telephone. The interviews had a median duration of 11.5 (range 5.5-14.5) minutes. In the interviews, 5 themes were identified within the 2 main topics (Table 4).

**Table 4:** Topics, themes, and quote examples from 6 patient interviews.

Topic and themes			Quotations
**Experienced effects of VRx^a^**			
	Visualization helps to perform exercises	*If you perform the exercises yourself, without virtual reality, you don’t know, for example, how long to inhale and exhale. So you perform the exercises, but I think you never really perform it as you should. You proceed much faster to the next step, so you will exhale much faster, and in the virtual reality exercises you see visually how fast you should inhale, how fast you should exhale, how long you need to keep going. You last less without the VR glasses, not because you can’t but because you think it’s enough like this.* [Woman, 56 years] *Also if you have pain, you don’t really think about it, you just do what you’re asked to because you want to score points. So you are completely into the game. You just do it and you have no sense of pain.* [Woman, 56 years] *… also movements you don’t think of yourself. I have a broken clavicle and ribs and then you are mostly focussing on that instead on moving, although there many more movements to make.* [Man, 49 years]	
	VRx immerses patients in a different world	*I really liked that. Sometimes it was very hectic in our room. At those times it was very nice to be able to cut yourself off.* [Woman, 64 years] *I really felt locked up in there. Yes, when I look around me, I had the feeling like I was somewhere underwater, I couldn’t really handle it.* [Man, 55 years]	
**Barriers and facilitators toward the use of VRx**			
	Easy to use VR^b^ technology is important	*I immediately understood, this way you turn it on. You put it on, you use it, you take it off and you turn it off. You ask someone to charge it. Well yes, that is very user friendly.* [Woman, 56 years] *In other words, I have to get it, I have to turn it on. Those are way more actions than when I quickly get that thing (points at incentive spirometer) […] And with those glasses.. I am not so technical, then you have to look it all up, you have to connect it, see which program, for me it was way more effort.* [Man, 55 years] *Well, at least that you have some kind of score, in which you can improve yourself.* [Man, 49 years]	
	Patients’ independence and hospital environment influence VR use	*When you have that thing (the VR headset) lying next to your bed, then it’s nothing much. But if you have to call someone every time to hand you that thing.. And when you have that thing lying in your bed, it is quite the box with equipment.* [Man, 62 years] *But well, those first two days there was so much going on, and the physiotherapist and the doctors and simultaneously the collapsed long and you know, it was just… I didn’t use it so much, let me put it this way, I would have liked to use it more.* [Woman, 64 years] *Let’s say we provide patients with a VR headset to take home […] at home you have more quiet. Like I said, in the hospital your life is organized by others.* [Man, 62 years]	
	Better integration in usual care might enhance efficacy	*…and yes, when the physiotherapist visited and I had to perform breathing exercises there, then I didn’t use the glassed right after. So I waited a while then.* [Woman, 64 years]	

^a^VRx: virtual reality therapy.

^b^VR: virtual reality.

#### Topic: Experienced Effects of VRx

##### Visualization Helps to Perform Exercises

Patients described that visualization of the exercises helped them to perform the exercises correctly and motivated to continue the exercises for a longer duration. The scoring system and gaming elements in the physical exercises motivated patients to move.

##### VRx Immerses Patients in a Different World

Patients experienced the immersiveness of VR as an analgesic, especially in the physical exercises. All patients experienced that VRx took them away from the hospital environment and immersed them in a different world. For most patients, this was a positive experience, and one felt locked up in the virtual world.

#### Topic: Barriers and Facilitators Toward the Use of VRx

##### Easy-to-Use VR Technology Is Important

The ease of use of the VR headset was the most important item for patients and was experienced differently. Several patients found the headset easy to use. However, some patients felt that using the headset took more effort compared with conventional exercises. For them, the experienced added value of the VR exercises did not outweigh the experienced additional effort. Integrated biofeedback in the VR exercises was mentioned to increase added value.

##### Patients’ Independence and Hospital Environment Influence VR Use

Patients who could use the VRx independently mentioned this as a facilitator to perform the exercises and appreciated to perform the VR exercises at their preferred time. Patients who could not use the headset independently, for example, because the nurse needed to handover the headset, experienced this as a barrier. The busy hospital ward was mentioned as a barrier. Conversely, some patients used the VRx to escape from this environment. Some patients would have liked to continue the VR exercises at home as additional support to the standard exercises at home.

##### Better Integration in Usual Care Might Enhance Efficacy

The software applications of the physical exercises could be improved by adapting to the hospital environment. Some exercises could not be adequately performed from a hospital bed, which caused frustration. Some patients felt that the exercises could be better integrated with physiotherapy care and mentioned that some caregivers seemed not familiar with the VRx.

### Premature Ending of Study

This study was ended prematurely due to an insufficient rate of accrual and operational futilities. At start of the study, the tail of the COVID-19 pandemic caused a delay in inclusion rate due to reduced bed capacity. After the first 3 months, the number of screened patients caught up with the planned screening numbers; however, the rate of accrual remained insufficient. Based on the actual recruitment rate, 5 additional years of recruitment would have been required to attain the necessary number of participants (n=126). This urged a critical appraisal of the inclusion procedure and consideration to expand the target patient population to other wards. Simultaneously, it was noticed during study conduct that adherence to the prescribed frequency of breathing and physical exercises was low in both the VR and control groups. This would result in a smaller effect of VRx than expected and a smaller difference with the control group. Consequently, the study would require a larger sample size to obtain a significant difference, while it would be questionable whether this difference would be clinically relevant. The low adherence in the control group prompted us to audit the protocol adherence at several wards with a comparable breathing exercise protocol. Altogether, we drew the conclusion that it would require an additional intervention to optimize protocol adherence, which was considered unrealistic and inappropriate in the current study.

## Discussion

### Principal Findings

The primary aim of this study was to assess the effectivity of VR breathing and physical exercises on the pulmonary recovery of patients with blunt chest trauma at the trauma ward. In this small sample, we observed no differences in final vital lung capacity between the VR group and control group. The pattern of vital lung capacity increase seemed to differ between groups, with the VR group reaching a higher capacity on day 3 than the control group. A similar pattern was observed for the independence in activities of daily living. Patients reported outcomes on quality of recovery and satisfaction did not differ between groups. Interviews demonstrated appreciation and potential of VR exercises, although several barriers were mentioned regarding feasibility and usefulness.

Despite a preceding audit of this patient group regarding eligibility and sufficient numbers, the trial was prematurely terminated due to enrollment failure. Enrollment failure is the main reason for termination in 60% of prematurely terminated trials [32,33]. A secondary reason for termination was poor adherence to the clinical guideline in both the control group and VR group with infrequent to no performance of breathing and physical exercises in both groups. We considered this a major drawback for continuation with this study protocol considering mostly unsupervised and self-administered exercises in both groups.

No conclusions can be drawn about the effectivity of VR exercises on pulmonary recovery of patients with blunt chest trauma due to the small obtained sample size. However, the results can be interpreted as those of a pilot randomized controlled trial, and several important lessons can be learned for future VR studies in similar and different contexts. First, the suitability of patients to use VR in a hospital (trauma) ward setting might be lower than generally expected. Before initiation of this trial, calculations were performed based on the hospital registry of the year 2020 of patients who were admitted to the hospital with blunt chest trauma for more than 24 hours and no ICU admission. We estimated that 60% (146/243) of these patients could be included based on a previous clinical study in our hospital [34]. However, only 21% (27/129) of these patients could be included in this trial. Main exclusion reasons were a headwound, inability to comprehend the study protocol (eg, language barrier), cognitive impairment, and delirium, together accounting for 44% (45/102) of patients excluded. Another 24% (24/102) declined to participate for various reasons such as feeling too sick, disinterested, and finding participation too burdensome. Especially, the proportion of patients excluded due reasons related to inability to use a VR headset was underestimated in designing this study. We did not investigate impact of age as barrier or facilitator for using VRx. However, contrary to what has been reported in literature, we did not observe any noticeable resistance or lack of motivation among older patients during the inclusion procedure or the intervention [35]. In literature, it is rarely described how many patients are eligible for VR use and the reasons for exclusion, such as inability to use a VR headset due to concomitant illness. For example, 10,776 patients were assessed for eligibility in a study of Wiechman et al [36] evaluating the effectiveness of VR for pain and anxiety management in trauma patients and only 184 (2%) were included. Spiegel et al [17] showed an inclusion rate of 23% in a general hospital population. In both studies, specific reasons for exclusion are not mentioned and are generically described as “not meeting inclusion criteria” and “declined to participate.” Future research should specifically report on the eligibility of patients and exclusion because of the inability to use VR.

Second, the setting in which VRx is applied should be thoroughly charted before conducting research or implementing VR. Although a physiotherapist and nurse were involved in the design of this study to ensure alignment with daily practice, adherence to protocolled exercises was unexpectedly low. A study of Martin et al [37] in postoperative patients showed that 26% of patients failed to use IS correctly and 38% denied ever using IS. This is consistent with our study in which on different days 33% (4/12) to 63% (5/8) of the patients in the control group used IS 2 times or less, which were likely the supervised exercises for measuring vital lung capacity. Martin et al [37] reported that following a brief educational intervention by a physician, 74% of patients were more confident to use IS during the remainder of their care. In our study, patients were encouraged and educated once daily by a physiotherapist to perform the breathing exercises, however adherence was still low. Literature shows that self-management and patient participation in the hospital can improve treatment adherence [38]. The intervention in this study was meant to engage patients in their treatment, however this did not result in improved treatment adherence. Generally, VR is considered appropriate as self-management tool for patients [39]. In this study, patients acknowledged this potential, but the intervention did not result in improved treatment adherence. It has been argued that VRx still requires professional guidance [40,41]. This professional guidance might even be of greater importance in a hospital environment, since patients have trouble using VRx independently due to illness and comorbidity. Nurses may play an important role in giving education, counseling, facilitating, and enhancing taking responsibility [42]. However, a high workload and contradicting patient expectations are factors that complicate patient engagement [43,44]. The low adherence in our study might imply that the hospital environment, including patients and caregivers, needs reorganization to allow the transferring of care possibilities to patients and supporting patients in acquiring the self-management skills needed for VRx [45]. Better education and training of both caregivers and patients could enhance successful implementation of interventions such as VR [40,45,46]. We cannot rule out that results are different for a setting with close monitoring by caregivers, for example, high care unit or highly disciplined patient groups such as injured military personnel.

Third, several identified barriers should be overcome to ensure successful deployment of VR on the hospital ward. The barriers were identified by interviewing patients focused on their user experiences. Over the last decade, collecting patient experiences has been emphasized as a starting point for improving patient care in general [47]. This study illustrates how experiences can reshape new innovations like VRx for blunt chest trauma. Patients mentioned the perceived usefulness relative to the system usability as important reason for low adherence and as a main barrier to self-managing the HMD and the different apps. As described in the Technology Acceptance Model designed by Sagnier et al [48], the intention to use a given technology is predicted by the perceived usefulness and the perceived ease of use. It was clear from reported experiences that the perceived usefulness did not outweigh the perceived ease of use in the interview group. Although many feasibility studies report on the acceptability of VRx, a systematic analysis of the acceptance of VR is rarely performed [48]. Furthermore, in feasibility studies, acceptability is defined by a variety of different outcome variables such as a sense of involvement, comfort, wish to use VR again in the future, withdrawal from study, and satisfaction [49-52]. A systematic analysis, for example, using the Technology Acceptance Model, may add to the knowledge derived from pilot and feasibility studies in medical VR. Another barrier was the busy hospital ward, albeit that some purposively took on the VR device to escape from the busy environment and pursue quietness and privacy. A similar finding was reported for VR postoperative pain management [53]. The results underline the different values of patients regarding the use of a digital technology and prompt the alignment of outcome measures regarded as relevant in the design and evaluation of a VR intervention [54,55].

The relevance of this study lies in underlying causes for early termination, the critical appraisal of the study setting and standard treatment in the control group, and the yield of the patient reported experiences from the interviews. Main lesson was the misassumption that patients adhere to the instructions in the hospital protocol for breathing and physical exercises and that physiotherapists, nurses, and physicians check protocol compliance. A preceding pilot study might have exposed inadequate compliance as well as the overestimation of eligible patients. However, we doubt such a pilot study would have revealed the barriers regarding integration of VR in the clinical workflow and the controversies in perceived usefulness between patients.

### Recommendations

Several recommendations can be made for research and implementation of clinical VR from the appraisal of our findings. First, adherence to study protocols should be charted before designing a VR clinical trial. This may be accomplished by “shadowing” clinician-patient interaction and patient’s functioning and well-being [56]. Simultaneously, data can be obtained for integration of VR treatment in the daily workflow and additional training of staff [40,46]. Second, individual patient reported experiences and values need to be prioritized in evaluating VR acceptance, usability, and effectiveness [53]. In line, we recommend to perform a systematic analysis on the acceptance before pilot or main effectiveness studies [48]. Third, eligibility of patients and exclusion of patients due to the inability to use VRx should be routinely reported.

### Conclusion

This clinical trial of self-administered VR treatment for blunt chest trauma had to be terminated prematurely due to enrollment failure and limited protocol compliance to breathing and physical exercises in both groups. Suitability of trauma patients to use VRx at a hospital ward was overestimated despite previous audit of potentially eligible study participants. Hospital setting, standard care, and patients’ perceptions of VR treatment seem important determinants for success in clinical VR research.

## Appendix

Multimedia Appendix 1 CONSORT (Consolidated Standards of Reporting Trials) checklist.

Multimedia Appendix 2 Schedule of study procedures and measurements.

Multimedia Appendix 3 Outcome variables per day for patients who completed three or more study days.

Multimedia Appendix 4 Analgesics use on consecutive study days for patients who completed three or more study days, presented as number (percentage).

## Abbreviations

CONSORT: Consolidated Standards of Reporting Trials
HMD: head-mounted display
ICU: intensive care unit
IS: incentive spirometer
MTSQ: modified treatment satisfaction questionnaire
PDL: powerlessness in daily living
QoR-15: Quality of Recovery-15 questionnaire
VAS: visual analog scale
VR: virtual reality
VRx: virtual reality therapy

## Data Availability

The datasets generated during and/or analyzed during the current study are available from the corresponding author on reasonable request.
