# Supplementary material for: Pulmonary and Physical Virtual Reality Exercises for Patients With Blunt Chest Trauma: Randomized Clinical Trial
Source: JMIR Serious Games. 2024 Dec 9;12:e54389. doi: 10.2196/54389 (PMC11667138; doi:10.2196/54389)
Supplement: Multimedia Appendix 2 [file games_v12i1e54389_app2.pdf]

Appendix 2. Schedule of study procedures and measurements

Protocol Activities	Day 1 after admission	Day 2-4	Day 5	Day 5 - discharge
Recruitment	X			
Verbal and written information	X			
Informed consent	X			
Explanation and training session	X			
Patient characteristics	X			
Incentive spirometry	X	X (2x daily)	X	
Wearable activity monitor	X (installing the wearable)	X	X (reading out the data)	
Intervention (intervention group)		X	X	
VAS pain score [a]	X	X	X	
Clinical outcomes: length of hospital stay, complications, transfer to ICU [b]				X
Analgesic use, incl. escape medication	X	X	X	
PDL-score [c]	X	X	X	
Quality of recovery		X	X	
Treatment satisfaction (questionnaire & interview)			X	
Safety		X	X	

[a] VAS: Visual Analogue Scale

[b] ICU: Intensive Care Unit

[c] PDL: Powerlessness in Daily Living questionnaire
