# Supplementary material for: Pulmonary and Physical Virtual Reality Exercises for Patients With Blunt Chest Trauma: Randomized Clinical Trial
Source: JMIR Serious Games. 2024 Dec 9;12:e54389. doi: 10.2196/54389 (PMC11667138; doi:10.2196/54389)
Supplement: Multimedia Appendix 3 [file games_v12i1e54389_app3.pdf]

Appendix 3. Outcome variables per day for patients who completed three or more study days.

		**Baseline (day 1)**	**Day 2**	**Day 3**	**Day 4**	**Day 5**
Incentive spirometry in mL, mean (SD)	VR	N=7	N=6	N=6	N=7	N=4
		1564 (511)	1730 (450)	2042 (303)	1929 (499)	1781 (504)
	Control	N= 11	N=12	N=12	N=10	N=6
		1325 (478)	1620 (519)	1665 (560)	1889 (447)	1950 (552)
Time standing or walking, %, mean (SD)	VR	-	N=3	N=4	N=3	N=3
		-	4 (2)	4 (4)	3 (2)	3 (2)
	Control	-	N=6	N=8	N=6	N=4
		-	3 (1)	5 (5)	4 (3)	3 (3)
Time sitting (incl. in bed), %, means (SD)	VR	-	N=3	N=4	N=3	N=3
		-	25 (27)	70 (41)	61 (41)	59 (38)
	Control	-	N=6	N=8	N=6	N=4
		-	58 (30)	55 (28)	62 (28)	48 (37)
Time lying, %, mean (SD)	VR	-	N=3	N=4	N=3	N=3
		-	70 (26)	27 (27)	36 (46)	38 (36)
	Control	-	N=6	N=8	N=6	N=4
		-	38 (30)	39 (25)	35 (26)	49 (36)
VAS pain[a] (0-100), median (Q1;Q3)	VR	N=7	N=6	N=6	N=5	N=5
		30 (18;56)	19 (9;49)	17 (4;27)	35 (12;46)	8 (2;59)
	Control	N=12	N=12	N=12	N=10	N=7
		47 (7;70)	16 (5;46)	16 (4;51)	8 (3;26)	12 (2;50)
PDL[b] (0-21), median (Q1;Q3)	VR	N=5	N=6	N=3	N=4	N=4
		9 (8;12)	6 (4;12)	5 (4;5)	7 (2;10)	10 (2;13)
	Control	N=10	N=8	N=10	N=8	N=7
		11 (4;15)	6 (1;11)	4 (0;13)	5 (1;13)	7 (2;13)
QoR[c] (0-150), median (Q1;Q3)	VR	-	N=6	N=3	N=4	N=4
		-	82 (73;102)	87 (59;87)	81 (57;111)	108 (67;110)
	Control	-	N=10	N=9	N=9	N=5
		-	89 (82;120)	83 (76;97)	99 (89;111)	85 (69;96)

[a] VAS: Visual Analogue Scale

[b] PDL: Powerlessness in Daily Living questionnaire

[c] QoR: Quality of Recovery questionnaire
