# Supplementary material for: Pulmonary and Physical Virtual Reality Exercises for Patients With Blunt Chest Trauma: Randomized Clinical Trial
Source: JMIR Serious Games. 2024 Dec 9;12:e54389. doi: 10.2196/54389 (PMC11667138; doi:10.2196/54389)
Supplement: Multimedia Appendix 4 [file games_v12i1e54389_app4.pdf]

Appendix 4. Analgesic use on consecutive study days for patients who completed three or more study days, presented as number (percentage).

	Day 1		Day2		Day 3		Day 4		Day 5	
	Control N=12	VR N=7	Control N=12	VR N=7	Control N=12	VR N=7	Control N=10	VR N=6	Control N=9	VR N=5
Non-opioids	12 (100)	7 (100)	12 (100)	7 (100)	12 (100)	7 (100)	10 (100)	6 (100)	8 (92)	5 (100)
Opioids	11 (92)	7 (100)	10 (83)	6 (86)	12 (100)	7 (100)	9 (90)	7 (100)	7 (78)	7 (100)
Epidural	2 (17)	2 (29)	2 (17)	2 (29)	2 (17)	2 (29)	2 (20)	2 (33)	2 (25)	2 (40)
PCIA	5 (42)	4 (57)	5 (42)	3 (43)	4 (33)	3 (43)	1 (10)	1 (17)	1 (12.5)	1 (20)
